# Recurrent Ischaemic Stroke in a Patient With Left Ventricular Thrombus Despite Apixaban: Successful Transition to Warfarin

**DOI:** 10.7759/cureus.94691

**Published:** 2025-10-16

**Authors:** Mohd Imran Patel, Sindhuja Kannan, Riya M Jacob

**Affiliations:** 1 Internal Medicine, Prince Charles Hospital, Merthyr Tydfyl, GBR

**Keywords:** anticoagulation, apixaban, case report, left ventricular thrombus, low ejection fraction, stroke, warfarin

## Abstract

Left ventricular (LV) thrombus is a well-recognised source of cardioembolic stroke, especially in patients with severely reduced LV ejection fraction (LVEF). Anticoagulation is the mainstay of therapy, but the optimal agent remains debated. We report the case of a 68-year-old man with an LV thrombus who suffered recurrent ischemic strokes while on apixaban, with magnetic resonance imaging (MRI) of the brain demonstrating acute infarcts in the left thalamocapsular and occipitotemporal regions. Following multidisciplinary team review, he was transitioned to warfarin with low-molecular-weight heparin (LMWH) bridging, after which he achieved therapeutic international normalised ratio (INR) stability and experienced no further clinically apparent embolic events. This case underscores the ongoing embolic risk associated with LV thrombus despite direct oral anticoagulant (DOAC) therapy, the importance of LMWH bridging when initiating warfarin, and the value of multidisciplinary management in optimising outcomes.

## Introduction

Cardioembolic stroke accounts for approximately 20-30% of all ischemic strokes, with left ventricular (LV) thrombus representing a significant but often underdiagnosed aetiology [1]. The risk of thromboembolism is highest in the early weeks following a large anterior myocardial infarction and in patients with severe LV systolic dysfunction [2]. Anticoagulation remains the cornerstone of therapy; however, recurrent embolic events may occur, particularly in the presence of large or mobile thrombi [3].

Vitamin K antagonists (VKAs), such as warfarin, have historically been the standard of care, with therapeutic anticoagulation (international normalised ratio (INR) 2.0-3.0) maintained for three to six months and guided by serial imaging [3]. In recent years, direct oral anticoagulants (DOACs) - a class of oral agents that directly inhibit thrombin or factor Xa, including apixaban, rivaroxaban and dabigatran - have emerged as attractive alternatives due to predictable pharmacokinetics and absence of routine monitoring requirements. Although early studies and case series suggest comparable efficacy to VKAs, the overall evidence remains limited and inconclusive [1,2,4].

This case is reported to highlight a clinically significant failure of apixaban in a patient with a documented LV thrombus who experienced embolic events despite being on apixaban, but subsequently stabilised following transition to warfarin with low-molecular-weight heparin (LMWH) bridging. This case highlights the ongoing risk of embolisation in LV thrombus and underscores the importance of individualised anticoagulant selection, vigilant follow-up, and multidisciplinary management.

## Case presentation

A 68-year-old male with a past medical history of hypertension, asthma/chronic obstructive pulmonary disease (COPD), epilepsy, and secondary polycythaemia, and a history of smoking since the age of 15 years, presented to the Accident and Emergency (A&E) Department in early April 2025 with progressive shortness of breath and bilateral pedal oedema. Transthoracic echocardiography performed during this admission revealed a LV thrombus and a severely reduced ejection fraction (EF) of 20-25%. He was commenced on apixaban and subsequently discharged home.

In late April 2025, the patient re-presented to A&E with increasing confusion, amnesia, and worsening dyspnoea. A computed tomography (CT) scan of the head was normal, while a CT pulmonary angiogram (CTPA) excluded pulmonary embolism but demonstrated a persistent 17 mm LV thrombus. As he was already anticoagulated with apixaban, no changes were made to his treatment, and he was reassured by the attending consultant before being discharged with outpatient follow-up.

**Figure 1:**
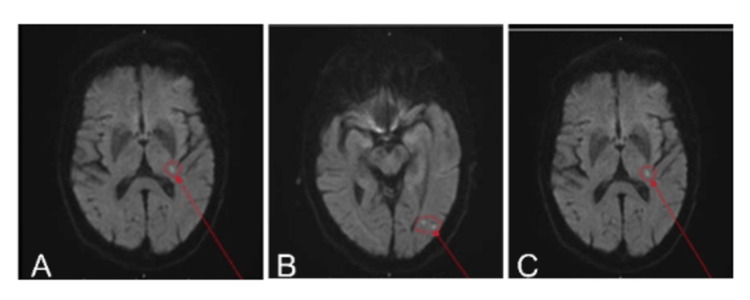
MRI brain images showing infarct regions. (A) Axial fluid-attenuated inversion recovery (FLAIR) highlighting periventricular lesion (circled and arrowed). (B) Axial FLAIR showing left occipitotemporal lesion (circled and arrowed). (C) Axial diffusion-weighted image demonstrating left thalamocapsular infarct (arrow). (D) Blank panel placeholder. The circle and arrow show the infarct.

In mid-July 2025, the patient was found at home by emergency services after a long period of lying. He had been incontinent of faeces and was noted to have left-sided arm and leg weakness, expressive dysphasia, and left-sided neglect. On admission, CT head, CT carotid angiogram, and CT intracranial angiogram were all unremarkable. Given the strong clinical suspicion of stroke despite negative imaging, his case was reviewed by the stroke consultant, who advised discontinuing apixaban and commencing aspirin 300 mg while awaiting magnetic resonance imaging (MRI) of the brain. MRI subsequently demonstrated tiny acute infarcts involving the left thalamic-capsular region and the left occipitotemporal region (Figure 1).

Despite the initiation of aspirin, the patient developed a further episode of stroke within a week. His case was discussed in a multidisciplinary team (MDT) meeting involving the stroke consultant, a haematologist, and an acute medicine consultant. The consensus was to initiate warfarin therapy, with bridging anticoagulation using therapeutic LMWH (enoxaparin) until the INR reached the target therapeutic range. Once stable, the patient was discharged home on warfarin and scheduled for regular INR monitoring at the anticoagulation clinic.

During follow-up, the patient maintained therapeutic INR levels, and no further episodes of stroke were reported over the subsequent months.

The sequence of clinical events, corresponding investigations, and management decisions is summarised in Table 1.

**Table 1:** Timeline of events. A&E - accident and emergency; CTA - computed tomography angiography; CTPA - computed tomography pulmonary angiogram; DOAC - direct oral anticoagulant; EF - ejection fraction; INR - international normalised ratio; LMWH - low-molecular-weight heparin; LV - left ventricle; MDT - multidisciplinary team; MRI - magnetic resonance imaging; PE - pulmonary embolus

Date	Event	Findings/Management
Early April 2025	First A&E admission with dyspnoea and oedema	Echo: LV thrombus, EF 20-25%. Started on apixaban.
Late April 2025	Re-presented with confusion and amnesia	CT head: normal. CTPA: no PE, persistent 17 mm LV thrombus. Continued on apixaban.
Mid-July 2025	Found at home after a long period of lying with left-sided weakness, dysphasia, neglect	CT head/CTA: normal. DOAC stopped, aspirin 300 mg started.
Mid-July 2025 (MRI)	MRI brain	Acute infarcts: left thalamic-capsular and occipitotemporal regions (Figure 1)
Late July 2025	Recurrent stroke on aspirin	MDT decision: switch to warfarin with LMWH bridging.
August 2025 onward	INR clinic follow-up	INR therapeutic and stable. No further strokes.

## Discussion

LV thrombus is a well-recognised cause of cardioembolic stroke. DOACs have emerged as convenient alternatives to VKAs for several thromboembolic conditions; however, their use in LV thrombus remains off-label, and the supporting evidence is limited and inconsistent [1-4]. Observational studies suggest that DOACs may achieve comparable thrombus resolution and embolic outcomes to VKAs [2,3], but guideline consensus continues to favour warfarin in high-risk patients, particularly those with severe LV systolic dysfunction or large thrombus burden [1,4]. In this patient, embolic events occurred despite ongoing apixaban therapy, highlighting the persistent risk of thromboembolism in patients with severe LV systolic dysfunction and substantial thrombus burden. Contributing factors may have included a hypercoagulable state related to secondary polycythaemia, although adherence, dosing, or potential drug interactions could not be fully ascertained. The patient was prescribed apixaban 5 mg twice daily, the therapeutic dose for stroke treatment and the patient was confirmed to be fully compliant based on pharmacy refill records, inpatient medication charts and administration charts by nurses. Patient renal and hepatic function remained within normal limits throughout the admissions, ruling out underexposure to pharmacokinetic variability. He was not receiving any concurrent drugs known to affect apixaban metabolism, such as strong CYP3A4 or P-glycoprotein inducers or inhibitors. These collectively strengthen the likelihood that the recurrent embolic events represented a true treatment failure rather than underdosing or non-adherence.

Following transition to warfarin, the patient's INR was titrated to a target range of 2.0 to 3.0, reaching therapeutic levels within five days under LMWH bridging. Warfarin initiation alone may expose patients to a transient procoagulant state. Bridging with LMWH provides continuous anticoagulation until the INR reaches the therapeutic range [5]. In this patient, the transition to warfarin with LMWH bridging successfully prevented further embolic events, demonstrating the clinical utility of this strategy. Similar approaches have been recommended in contemporary reviews of LV thrombus management, which emphasise that careful monitoring during the initial phase of VKA therapy is critical to reduce the risk of recurrent events [6,7].

Multidisciplinary input was instrumental in optimising patient outcomes. Stroke physicians, haematologists, and acute medicine specialists collaborated to tailor anticoagulation after recurrent events, highlighting the importance of team-based decision-making in complex cases [8]. The combination of expert clinical judgment, appropriate imaging follow-up, and careful monitoring of therapeutic anticoagulation contributed to the patient’s subsequent stability.

Overall, this case reinforces that although DOACs may offer practical advantages, warfarin remains the standard of care for patients with LV thrombus who are at high risk for recurrent embolic events. Clinicians should consider individual thrombotic risk factors, ensure rigorous therapeutic monitoring, and involve MDTs when planning and adjusting anticoagulation therapy [1-8].

## Conclusions

This case illustrates the challenges of managing LV thrombus in patients with severe LV systolic dysfunction and additional prothrombotic risk factors. Recurrent embolic events occurred despite DOAC therapy, and switching to warfarin with LMWH bridging was associated with clinical stabilisation and prevention of further strokes.

Clinicians should individualise anticoagulation strategies for LV thrombus, maintain vigilant monitoring during transitions between agents, and engage MDTs when planning therapy. Serial imaging and careful INR management remain essential to reduce recurrent cardioembolic events.
